# Carriage burden, multiple colonization and antibiotic pressure promote emergence of resistant vaccine escape pneumococci

**DOI:** 10.1098/rstb.2014.0342

**Published:** 2015-06-05

**Authors:** Patrick K. Mitchell, Marc Lipsitch, William P. Hanage

**Affiliations:** 1 Center for Communicable Disease Dynamics, Department of Epidemiology, Harvard T.H. Chan School of Public Health, Boston, MA 02115, USA; 2 Department of Immunology and Infectious Diseases, Harvard T.H. Chan School of Public Health, Boston, MA 02115, USA

**Keywords:** *Streptococcus pneumoniae*, vaccination, emergence, model, resistance

## Abstract

Pneumococcal conjugate vaccines target the limited subset of the more than 90 known serotypes of *Streptococcus pneumoniae* responsible for the greatest burden of pneumococcal disease and antibiotic resistance. Following the introduction of these vaccines, serotypes not targeted were able to expand and resistance became more common within these types. Here we use a stochastic dynamic model of pediatric pneumococcal carriage to evaluate potential influences on the emergence of new resistant lineages following the introduction of a vaccine targeting more common resistant types. Antibiotic pressure was the strongest driver, with no emergence at low levels and universal emergence at high levels. At intermediate levels of antibiotic pressure, higher carriage burden and a greater degree of dual carriage promoted emergence. This may have implications for current plans to introduce childhood pneumococcal vaccination in several high-burden countries.

## 1. Introduction

*Streptococcus pneumoniae* (the pneumococcus) is a common, widespread bacterial commensal and pathogen. Worldwide, it is estimated to colonize as many as 40–60% of young children [1]. While colonization most frequently results in asymptomatic carriage, *S. pneumoniae* is still responsible for a substantial burden of disease. In 2000, there were an estimated 14.5 million episodes of severe pneumococcal disease resulting in 826 000 deaths in children under the age of five. Excluding pneumococcal deaths in HIV-positive children, this accounts for approximately 11% of under-5 mortality [2].

Pneumococcal strains are classified into more than 90 different serotypes based on the antibody reactivity and chemical structure of the polysaccharide capsule, the major surface antigen of pneumococci. Pneumococcal conjugate vaccines (PCVs) have proved effective in reducing the populations of pneumococci bearing vaccine type (VT) capsules, but only target a handful of serotypes. PCV use has been followed by the emergence of strains of non-vaccine serotype (NVT) in carriage [3,4] and, to a lesser degree, disease [5]. Among these are some VT lineages which have evaded the vaccine by acquiring an NVT capsule by recombination, a frequent process in pneumococci [6]. Such ‘capsule switch’ variants have successfully spread across the USA and internationally following the adoption of the heptavalent PCV (PCV7) in 2000 [6,7]. In addition to genes involved in capsule biosynthesis, other genes primarily found in VT lineages have the potential to enter NVT lineages through recombination [8]. In the absence of vaccination, these genes may be rare or absent in NVT lineages. Whether they first appear in NVT lineages before or after vaccine introduction, these genes may help the NVT lineages in which they are found expand into niches cleared of VT lineages. Genes that affect antigenicity, pathogenicity and antibiotic resistance are of particular concern given the relevance of these phenotypes to human disease. These genes have also shown a high frequency of observed recombination, reflecting one or both of two factors: strong selective pressure favouring recombinants and/or intrinsically higher rates of genetic recombination [6,9].

The emergence of new vaccine escape variants, particularly those exhibiting high-level antibiotic resistance, will continue to cause concern as PCV use expands. According to the March 2014, Vaccine Information Management System (VIMS) Global Vaccine Introduction Report [10], 39 countries currently have plans to introduce a PCV. Of the countries planning introduction, 22 are classified as middle income and 11 as low income. The epidemiology of *S. pneumoniae* in such settings is highly variable and can be quite different from that in higher income settings [11,12]. Additionally, antibiotic usage can differ sharply between countries [13]. As such, the effect of PCV introduction on the emergence of resistant vaccine escape variants may differ from what has been seen thus far.

Of the major lineages that emerged following the introduction of PCV7, most appear to have been present at a low frequency prior to vaccination [6,8,14]. As such, we sought to evaluate factors that may influence the emergence of a rare antibiotic-resistant pneumococcal lineage following the introduction of vaccination targeting more common resistant types. Here, we employ a stochastic dynamic model of pneumococcal carriage to assess the role of antibiotic pressure, carriage burden, and multiple colonization in promoting this emergence.

## 2. Model structure

**Figure 1.**
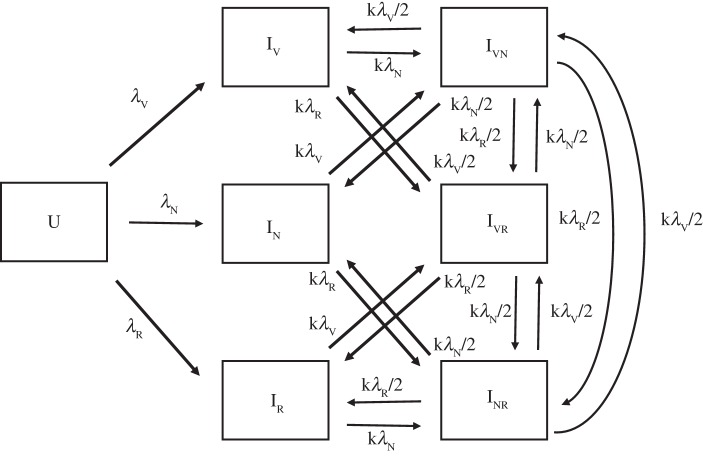
Box diagram of model. Each compartment is further subdivided into vaccinated and unvaccinated populations. The U compartment is uncolonized and subscripts in each colonized (I) compartment indicate the colonizing strains (V, VT-R; N, NVT-S; R, NVT-R). In the vaccinated population, *λ*_V_ is replaced by *vλ*_V_. Entry occurs into the U compartment and exit out of the model population occurs at the same rate from all compartments (not shown). All I compartments recover into the U compartment at rate *d*.

Acquisition and clearance of *S. pneumoniae* carriage is simulated here using a three-strain SIS model designed to avoid implicit promotion of coexistence by limiting competition between strains [15]. A condensed model schematic is shown in figure 1. The model uses an open population in which people exit the population from each compartment at rate *u*, here set at 1/60 month^−1^. People enter the population at the same rate multiplied by the initial population size in order to produce a roughly stable population size covering a 5 year age range.

Individuals may be colonized either by a single strain or any combination of two strains. Full details of each compartment are given in table 1 and parameters are described in table 2. Dual colonization with a single strain is treated as being identical to single colonization with that strain. Those currently colonized have their susceptibility to acquiring another strain reduced by a relative susceptibility factor *k*. If an individual colonized by two strains acquires a third, it is equally probable that either of the two original colonizing strains will be replaced by the new one. An important feature of the model structure is that individuals in the dual-colonized compartment can acquire new colonization with one of the two strains they are already carrying. If the new colonizing strain is the same as that which it is replacing, the individual remains in the same compartment, colonized by the same two strains; if it replaces the other, such that the host is now ‘dual-colonized’ with the same strain, the host is moved into the appropriate single carriage compartment. Clearance of colonization occurs at the same rate *d* from all colonized compartments. Each compartment in the model is duplicated to distinguish between vaccinated and unvaccinated individuals. A fraction *f* of new entrants, here set to 90%, are vaccinated at birth and thus become part of the vaccinated population, while the rest enter the fully susceptible population.

**Table 1.** Description of compartments.

compartment		
unvaccinated	vaccinated	description
U	V	uncolonized
I_VS_	I_VV_	colonized with VT-R strain only
I_NS_	I_NV_	colonized with NVT-S strain only
I_RS_	I_RV_	colonized with NVT-R strain only
I_VNS_	I_VNV_	colonized with both VT-R and NVT-S strains
I_VRS_	I_VRV_	colonized with both VT-R and NVT-R strains
I_NRS_	I_NRV_	colonized with both NVT-S and NVT-R strains

**Table 2.** Description of parameters.

parameter	value	description
*β*	1.5–2.5 × 10^−5^	transmission parameter—corresponds to *R*_0_ = 1.5–2.5, w. one month duration and total population = 10^5^ [11,12,16]
*d*	one month^−1^	rate of clearance, corresponds to one month duration of carriage
*u*	1/60 month^−1^	entry/exit rate
*a*	1.0–1.05	transmission advantage of VT and recombinant strains
*k*	0–0.5	relative susceptibility of those already colonized (0 = no dual colonization) [17–21]
*v*	0.5	reduction of transmissibility of VT strain to vaccinated individuals [22]
*f*	0.9	fraction of new entrants into the population that are vaccinated
λ_V_	*a*β(I_VS_ + I_VV_ + 0.5(I_VNS_ + I_VNV_ + I_VRS_ + I_VRV_)	force of infection with VT-R strain in unvaccinated and vaccinated populations, respectively
	*va*β(I_VS_ + I_VV_ + 0.5(I_VNS_ + I_VNV_ + I_VRS_ + I_VRV_)	
λ_N_	β(I_NS_ + I_NV_ + 0.5(I_VNS_ + I_VNV_ + I_NRS_ + I_NRV_)	force of infection with NVT-S strain
λ_R_	*a*β(I_RS_ + I_RV_ + 0.5(I_VRS_ + I_VRV_ + I_NRS_ + I_NRV_)	force of infection with NVT-R strain

Each strain is defined by a combination of two characteristics. The first represents serotype, dichotomized into VT and NVT. Vaccinated individuals are protected from colonization with a VT strain by a factor *v*, set to produce a 50% reduction in susceptibility [22]. The other characteristic is a selective advantage conferred by antibiotic resistance implemented as a multiplicative increase *a* in the transmission parameter. This is meant to correspond to an increased availability of hosts to resistant strains, as a certain proportion of the population may be taking an antibiotic at any given time. We conceptualize this resistance trait as being conferred through large-scale genomic changes, such as mosaic penicillin binding proteins conferring β-lactam resistance or mobile elements introducing macrolide resistance genes [23], rather than being introduced by a single point mutation. As such, *de novo* generation of resistance is not included in this model.

Three combinations of these two traits are considered in the model. The first is an antibiotic-resistant VT strain (VT-R) and the second is an antibiotic-susceptible NVT strain (NVT-S). The former has a potential advantage in unvaccinated populations as a result of being antibiotic-resistant, while the latter is at an advantage in vaccinated populations due to its NVT capsule. The third strain combines the advantageous traits of the first two, being both antibiotic resistant and having an NVT capsule. The fourth possible combination, in which the strain would be inhibited by both vaccination and antibiotic use, is not considered as it would be quickly eliminated in the scenarios considered here.

The initial pneumococcal population is composed primarily of the VT-R and NVT-S strains in equal proportion. This is meant to reflect a hypothetical situation in which the vaccine being introduced targets the serotypes currently associated with the great majority of antibiotic resistance, similar to the situation when PCV7 was first introduced in the USA [24]. The starting prevalence of the NVT-R strain is set to 1% of the combined initial prevalence of the VT-R and NVT-S strains. This starting prevalence was chosen based on the well-documented emergence of serotype 19A ST320 pneumococci following the introduction of PCV7. In a study of the response of the pneumococcal population to vaccination, carriage isolates were collected from children attending Massachusetts primary care practices in 2001 and 2004. In the combined sample size of over 300, a single isolate was observed with the combination of serotype (19A) and sequence type (ST 320) that went on to become successful [25,26]. A true prevalence of 1% is near the upper bound of the prevalence in the underlying population that could be expected to produce this observation under the binomial distribution.

## 3. Model implementation

Using this model, we sought to compare the potential for emergence across a range of settings. The initial combined carriage prevalence of the VT-R and NVT-S strains was set to either 30%, 50% or 60% with corresponding baseline *R*_0_ values of approximately 1.5, 2.0 and 2.5 in order to simulate low, moderate and high burden settings [11,12]. The resistance advantage for the VT-R and NVT-R strains varied from 1.0 (selective neutrality) to 1.05 (5% advantage) to simulate increasing levels of antibiotic pressure.

A proportion of carriers are known to be colonized with multiple serotypes, suggestive of multiple acquisitions. Colonization with two (or, in rare cases, three) serotypes has been reported at levels consistent with those colonized having experienced an approximately 80% reduction in susceptibility to colonization with a new serotype, corresponding to a relative susceptibility of 20% when compared with uncolonized hosts [17,18]. Other studies have attempted to estimate the degree of competition in the acquisition of a new strain and found the relative susceptibility of those already colonized to range from less than 10% to nearly 50% [19–21]. To examine the impact of multiple carriage, the relative susceptibility of those currently colonized compared to those not currently colonized ranged from 0 (competitive exclusion with no dual colonization) to 50%.

Aside from this barrier to dual colonization, this model does not include any within-host competition and both strains in a dual colonized host are assumed to be equally likely to transmit. An alternative scenario in which the two resistant strains are given a lower relative fitness when compared with the sensitive strain, resulting in the resistant strains being less likely to transmit from a host also colonized with the sensitive strain, is considered in the electronic supplementary material.

The model was run as a series of stochastic simulations using the adaptive tau-leaping method implemented in R through the ‘adaptivetau’ package [27]. For all parameter sets, 50 realizations were conducted. The initial conditions of each simulation were meant to approximate pre-vaccine equilibrium conditions and the immediate introduction of vaccination. The combined starting prevalence of the VT-R and NVT-S strains was set to roughly the equilibrium prevalence predicted by the *R*_0_ used and the starting vaccine coverage was 90%, equal to the proportion of the incoming population vaccinated. The effect of lower or no vaccine coverage is evaluated in the electronic supplementary material, figures S1 and S2. Each simulation covered a 10 year timespan and had a starting population of 100 000 plus the starting number with the new strain. The final prevalence of the NVT-R strain was taken as the primary outcome and emergence was defined as a final NVT-R strain prevalence greater than 10%. Outcomes for the VT-R and NVT-S strains are detailed in the electronic supplementary material, figure S3.

## 4. Results

### (a) Effect of antibiotic pressure

**Figure 2.**
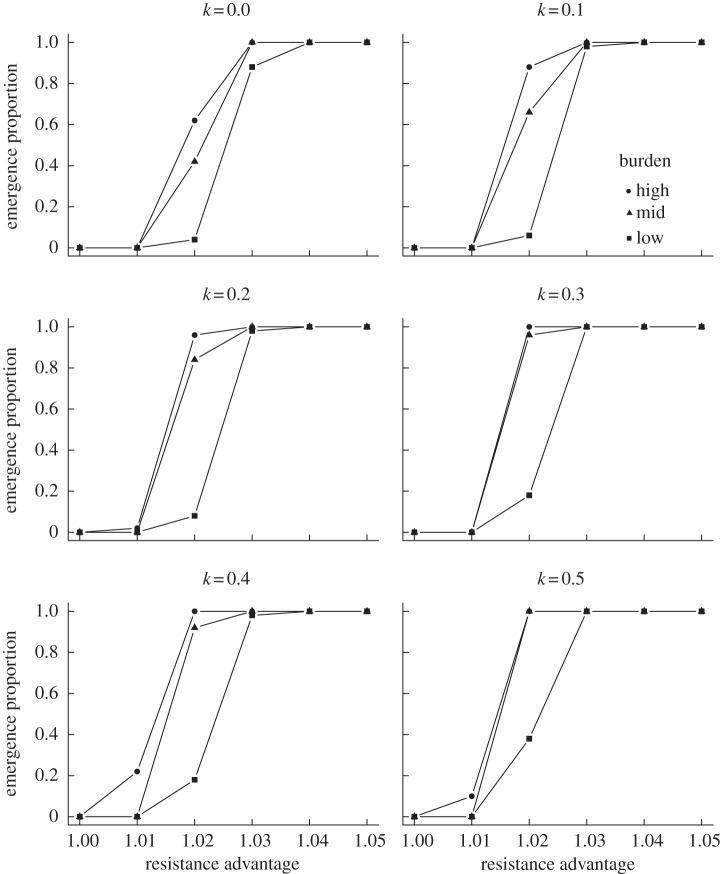
Proportion of realizations in which emergence (final NVT-R strain prevalence more than 10%) was observed by degree of antibiotic pressure. Each point represents a combination of antibiotic pressure (*x*-axis), carriage burden (lines) and relative acquisition rate *k* of dual versus single carriage (panels).

In order to assess the potential for emergence of a new resistant strain over a range of antibiotic pressures, simulations were conducted with the advantage parameter for the VT-R and NVT-R strains set between 1.0 and 1.05 in increments of 0.01. Varying the transmission advantage of the antibiotic-resistant strains had predictable effect on emergence. At lower values, the NVT-R strain was unable to overcome the greater initial prevalence of the NVT-S strain and could not colonize a substantial portion of the population within 10 years. Under neutral conditions no emergence was observed. With a 1% advantage, emergence was observed in a minority of realizations for the high-burden setting. At 3% and above, emergence was nearly universal, indicating that antibiotic use is a key determinant of the emergence of resistant lineages following PCV introduction. When the selective advantage due to antibiotic resistance was 2%, the potential for emergence varied by carriage burden and relative susceptibility to dual carriage (figure 2).

### (b) Greater emergence at higher carriage burden

As plans progress to introduce PCVs into new settings, it is worthwhile to consider the prospects for emergence across a range of carriage burdens reflecting the epidemiological situation in contexts other than the USA and Europe. To this end, the pneumococcal carriage prevalence was set to approximately 30, 50 or 60% through a combination of the starting prevalence and transmission parameter. As noted above, across carriage burdens, emergence occurred in all realizations when the selective advantage due to antibiotic resistance was 4% or greater, and emergence was never observed when there was no resistance advantage. At intermediate levels of antibiotic pressure, however, emergence was more likely in higher burden settings (figure 2). The NVT-R strain was also responsible for a higher proportion of colonizations in the higher burden settings (electronic supplementary material, figure S4). While the new strain was never completely absent by the end of the simulation, indicating that important changes are possible over a longer time frame than we consider here, these results suggest that emergence of new antibiotic-resistant lineages may occur more rapidly and with less antibiotic pressure in settings with higher pneumococcal burdens. When the relative fitness of resistant strains was low when compared with sensitive strains, however, this effect may be reversed (electronic supplementary material, figure S5).

### (c) Dual carriage promotes emergence

**Figure 3.**
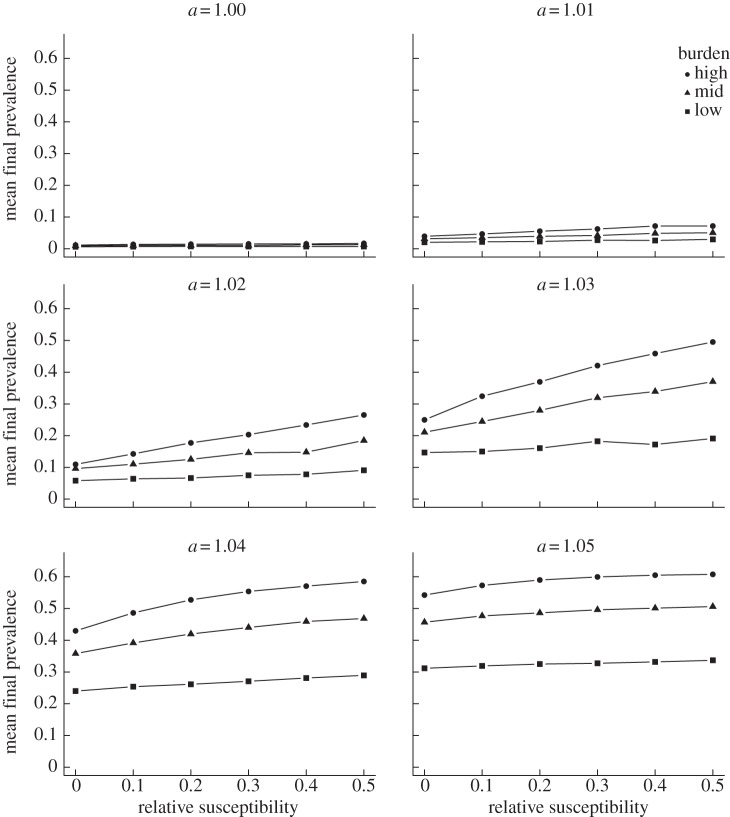
Effect of dual carriage on the final prevalence of the NVT-R strain. Each point represents the mean final prevalence of the NVT-R strain at a particular combination of dual carriage relative acquisition rate *k* (*x*-axis), carriage burden (lines) and antibiotic pressure (panels).

We evaluated the effect of competition and dual carriage by altering the relative susceptibility of those currently colonized compared to those not currently colonized between 0 (no dual carriage) and 0.5 in increments of 0.1. In general, increases in dual carriage allowed for greater spread of the NVT-R strain within the population. Across all scenarios considered, the mean final prevalence of the NVT-R strain was strongly correlated with relative susceptibility to dual carriage, though the effect was most pronounced at intermediate values for the resistance advantage (figure 3). When resistance conferred a fitness cost, however, increased dual carriage inhibited emergence of the NVT-R strain (electronic supplementary material, figure S5). Similar to the effect of carriage burden, the role of dual carriage was of secondary importance at high or low antibiotic pressures, as the new strain either approaches its maximum prevalence or is present at only very low levels, respectively.

## 5. Discussion

As PCVs are introduced into more immunization programmes, there will be continued alterations of the pneumococcal population. Here we evaluated several factors that may influence the emergence of previously unseen antibiotic-resistant lineages following the widespread introduction of pneumococcal vaccination. As would be expected, the degree of antibiotic pressure in the population is highly influential and appears to guarantee the emergence of new resistant types if sufficiently strong. When selection for antibiotic resistance was not as strong, increases in carriage burden and allowance for dual colonization also promoted emergence. These factors can both be seen as increasing the availability of new hosts, which are better exploited by the new resistant strain.

The observed effect that dual colonization promotes emergence suggests that intraspecific and intrahost competition may be important factors in determining the success of new pneumococcal variants. As this model simply dichotomizes pneumococci into VT and non-VT and does not consider any immunological factors beyond vaccination, it cannot fully capture these dynamics. Significant work has gone into modelling this type of competition in more detail using individual-based models [28,29]. While adding more realism in this respect would structurally alter the model, the qualitative findings would likely be similar, as the degree of antibiotic pressure was a much stronger driver of emergence. Additionally, previous models with differing structures have predicted expansion of rare NVT serotypes following vaccination yet did not consider resistance [30], and in another case the maintenance of penicillin resistance even after vaccination targeting all currently resistant serotypes in a setting with significant selection for resistance in a model that did not account for multiple carriage [16]. We have integrated these factors into a single model with the conclusion that antibiotic pressure is likely the most important driver of the emergence of resistant, NVT lineages.

Our findings also align with observed changes in the distribution of antibiotic resistance following the introduction of PCV7. Non-susceptibility to multiple classes of antibiotics rose in NVT pneumococci as they replaced VTs [31] and common lineages were displaced by more resistant clones within specific NVT serotypes. Examples of this phenomenon include sequence type (ST)320 replacing ST199 in serotype 19A [32] and CC176 being supplanted by ST386 in serotype 6C [33]. This combination of observed data and other modelling work lend credence to our finding that the expansion of a previously rare antibiotic-resistant lineage is probable following the introduction of a PCV targeting common resistant types. So long as vaccination targets only a subset of pneumococcal serotypes, antibiotic pressure will likely lead to the emergence of resistant lineages. This may be facilitated in settings that combine high or intermediate drug pressure with high carriage prevalence.

Despite this, conjugate vaccines have been and will continue to be a valuable tool for reducing the public health burden imposed by *S. pneumoniae*. Substantial reductions in pneumococcal disease, particularly due to pneumococci non-susceptible to penicillin and to multiple antibiotics, were observed in the aftermath of PCV7 [34]. This has occurred despite carriage prevalence remaining roughly constant [3,4], probably due to differences in invasive capacity between serotypes [35]. The strategy of vaccinating against the serotypes associated with the most worrisome pneumococcal lineages has indeed proved successful, but the plasticity of the pneumococcus necessitates continued vigilance to monitor for emerging variants [7,8,14,31–33].

## Supplementary Material

Supplementary Material
