# Supplementary material for: Carriage burden, multiple colonization and antibiotic pressure promote emergence of resistant vaccine escape pneumococci
Source: Philos Trans R Soc Lond B Biol Sci. 2015 Jun 5;370(1670):20140342. doi: 10.1098/rstb.2014.0342 (PMC4424439; doi:10.1098/rstb.2014.0342)
Supplement: Supplementary Material [file rstb20140342supp1.pdf]

Patrick K. Mitchell, Marc Lipsitch, William P. Hanage. 2015 Carriage Burden, Multiple Colonization, and Antibiotic Pressure Promote Emergence of Resistant Vaccine Escape Pneumococci. *Phil. Trans. R. Soc. B.* **370** doi: 10.1098/rstb.2014.0342

Effect of Lower Vaccine Coverage

In order to evaluate the extent to which our results would be altered by lower vaccine coverage, we ran five realizations with vaccine coverage reduced from 90% to 50% at each combination of carriage burden, antibiotic pressure, and dual carriage level. The mean final prevalence of the NVT-R strain was highly similar between simulations with 50 and 90% vaccine coverage for all parameter sets, indicating that high vaccine coverage is not necessary to reproduce the observed emergence pattern **[Figure S1]**. Vaccine coverage of 50% was also sufficient to eliminate the VT-R strain within 10 years in all simulations. With no vaccination, the NVT-R strain was never able to infect greater than 10% of the population. The VT-R and NVT-S strains infected similar proportions of the population with no vaccination and no resistance advantage, but as antibiotic pressure increased, the VT-R strain became dominant **[Figure S2]**.

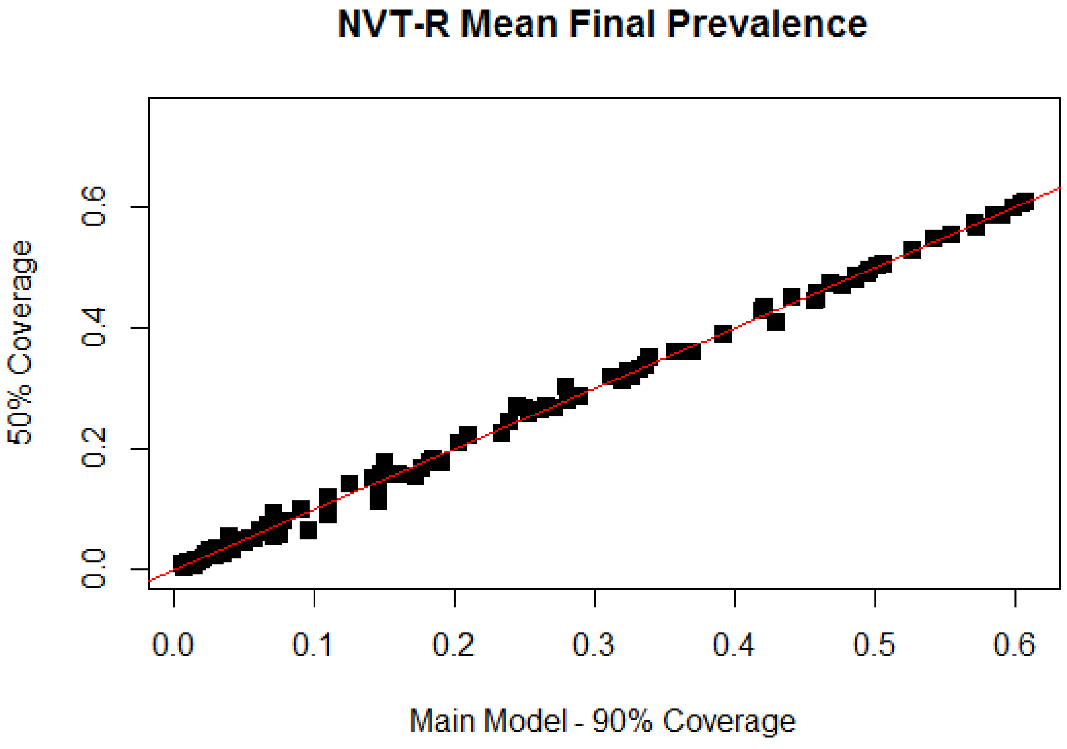

Figure S1: Comparison of final prevalence of NVT-R strain with 50% vaccine coverage to results from the main model with 90% coverage. Each point represents the mean final prevalence of the NVT-R strain at some combination of carriage burden, antibiotic pressure, and dual carriage level over 5 realizations and 50% coverage and 50 realizations at 90% coverage. The red line represents a perfect 1:1 correspondence between the mean final prevalence of the NVT-R strain between the two levels of vaccine coverage.

Patrick K. Mitchell, Marc Lipsitch, William P. Hanage. 2015 Carriage Burden, Multiple Colonization, and Antibiotic Pressure Promote Emergence of Resistant Vaccine Escape Pneumococci. *Phil. Trans. R. Soc. B.* **370** doi: 10.1098/rstb.2014.0342

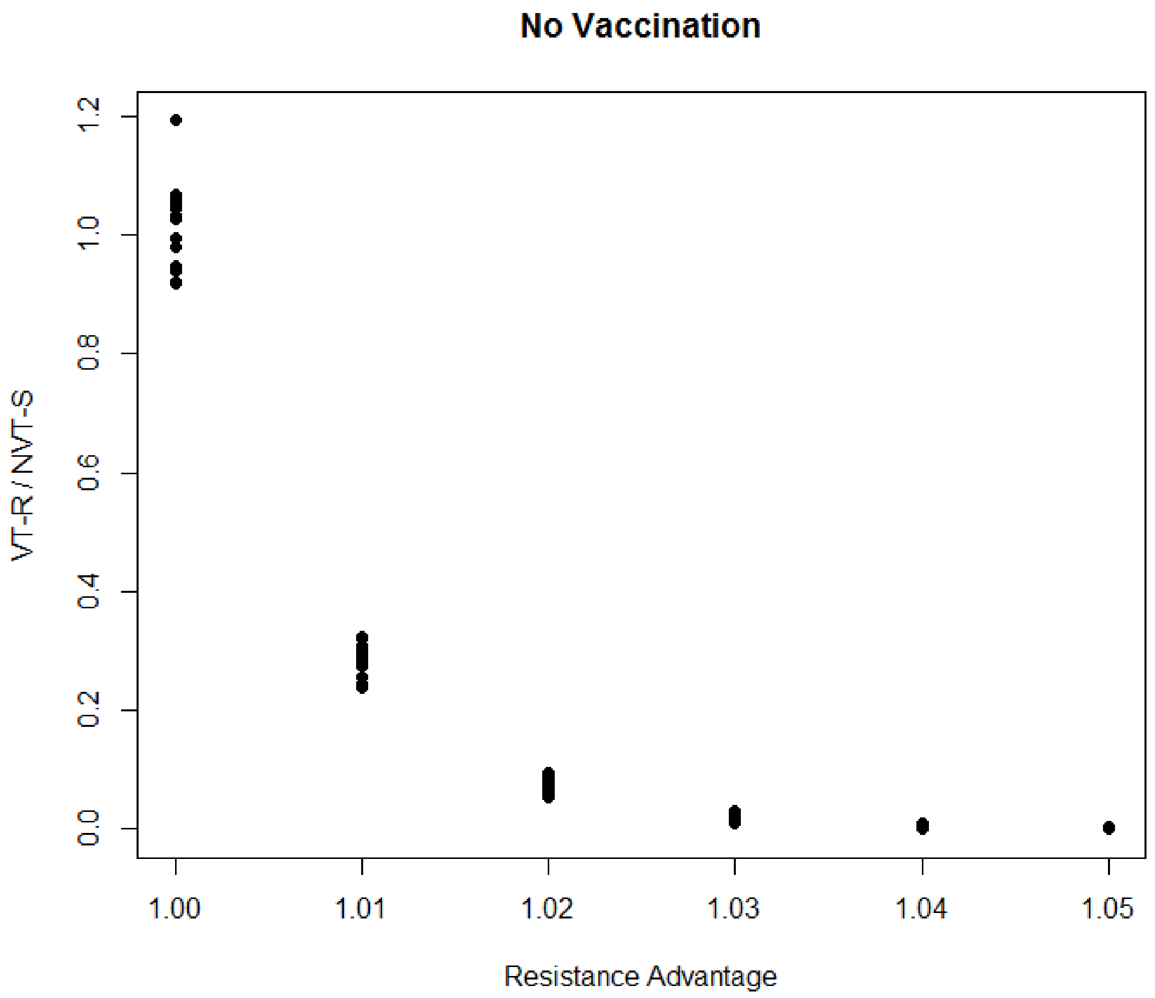

Figure S2: Ratio of the mean final prevalence of the VT-R and NVT-S strains across levels of antibiotic pressure in the absence of vaccination. Each point represents the mean outcome over five realizations at each combination of carriage burden, antibiotic pressure, and allowance for dual carriage.

Patrick K. Mitchell, Marc Lipsitch, William P. Hanage. 2015 Carriage Burden, Multiple Colonization, and Antibiotic Pressure Promote Emergence of Resistant Vaccine Escape Pneumococci. *Phil. Trans. R. Soc. B.* **370** doi: 10.1098/rstb.2014.0342

Relationship between Final Prevalences of Strains

An additional 5 realizations of the model with 90% vaccine coverage were run at each parameter set to track the mean final prevalence of each of the three strains as well as the proportion of the population uncolonized. The VT-R strain was completely eliminated from the population in all simulations. There was an inverse relationship between the mean final prevalence of the NVT-S and NVT-R strains, with the overall final prevalence remaining roughly constant across parameter sets **[Figure S3]**. This indicates that emergence of the NVT-R strain comes at the expense of the NVT-S strain, rather than increasing the overall carriage burden.

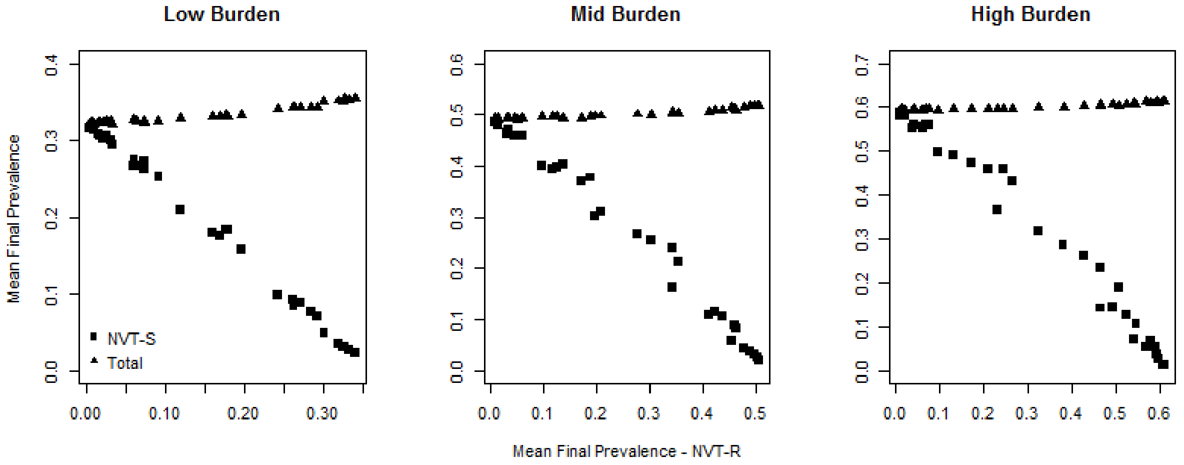

Figure S3: Total final prevalence and final prevalence of NVT-S strain in relation to final prevalence of NVT-R strain. Each point represents the mean results over five realizations at each combination of burden, antibiotic pressure, and dual carriage. The final prevalence of the NVT-S strain (squares) decreases as mean final prevalence of the NVT-R strain increases, while the total final prevalence (triangles) remains roughly constant.

Patrick K. Mitchell, Marc Lipsitch, William P. Hanage. 2015 Carriage Burden, Multiple Colonization, and Antibiotic Pressure Promote Emergence of Resistant Vaccine Escape Pneumococci. *Phil. Trans. R. Soc. B.* **370** doi: 10.1098/rstb.2014.0342

Effect of Carriage Burden on NVT-R Proportion

To evaluate whether the effect of higher carriage burden increasing emergence was solely due to a higher equilibrium prevalence, we compared the proportion of colonized carrying the NVT-R strain between carriage burdens. Using the same set of 5 realizations for each parameter set, the mean final prevalence of the NVT-R strain was divided by the overall mean final prevalence of colonization. At intermediate values of antibiotic pressure, the NVT-R strain was responsible for the greatest proportion of colonizations in the high burden setting and the least in the low burden setting **[Figure S4]**.

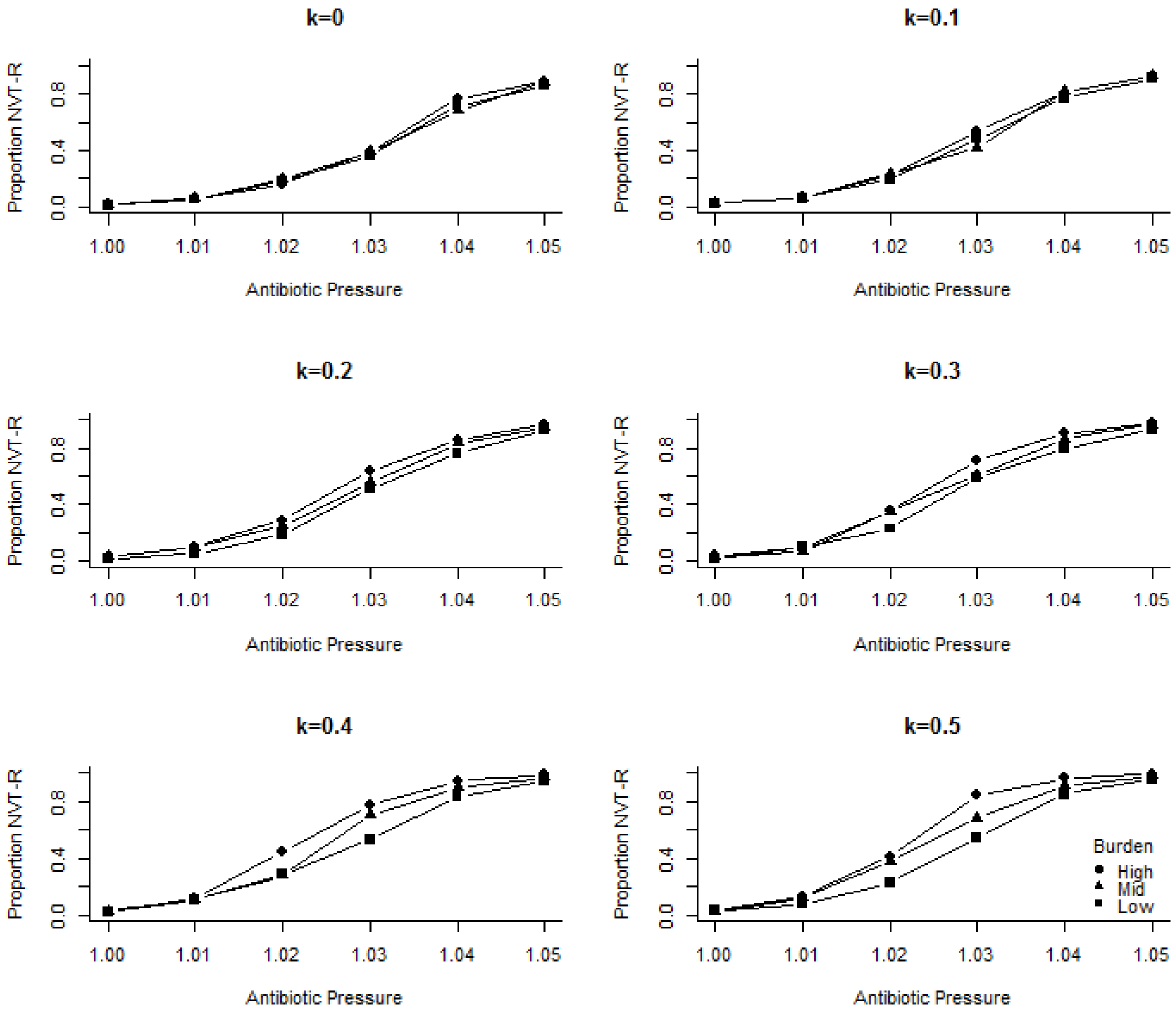

Figure S4: Proportion of the overall final prevalence due to the NVT-R strain. Each point represents the mean final prevalence of the NVT-R strain divided by the overall mean final prevalence over five realizations at each combination of burden, antibiotic pressure, and dual carriage.

Patrick K. Mitchell, Marc Lipsitch, William P. Hanage. 2015 Carriage Burden, Multiple Colonization, and Antibiotic Pressure Promote Emergence of Resistant Vaccine Escape Pneumococci. *Phil. Trans. R. Soc. B.* **370** doi: 10.1098/rstb.2014.0342

Decreased Relative Fitness Inhibits Emergence

Genetic changes encoding antibiotic resistance are typically thought to impose a fitness cost in antibiotic free environments. In order to explore how such a cost could affect the emergence of the NVT-R strain, we altered our model such that hosts colonized with both a susceptible and resistant strain could be more likely to transmit the susceptible strain than the resistant one. The relative fitness of the resistant strain was altered between 0.5 (resistant half as likely to transmit as sensitive) to 1.0 (equal probabilities, as in main model) in increments of 0.1. For each value for relative fitness, 5 realizations were conducted at each combination of carriage burden, antibiotic pressure, and dual carriage evaluated in the main model. As would be expected, lower relative fitness strongly inhibited the emergence of the NVT-R strain. This effect became more pronounced as the allowance for dual carriage increased and was less pronounced in the low carriage burden setting **[Figure S5]**.

Patrick K. Mitchell, Marc Lipsitch, William P. Hanage. 2015 Carriage Burden, Multiple Colonization, and Antibiotic Pressure Promote Emergence of Resistant Vaccine Escape Pneumococci. *Phil. Trans. R. Soc. B.* **370** doi: 10.1098/rstb.2014.0342

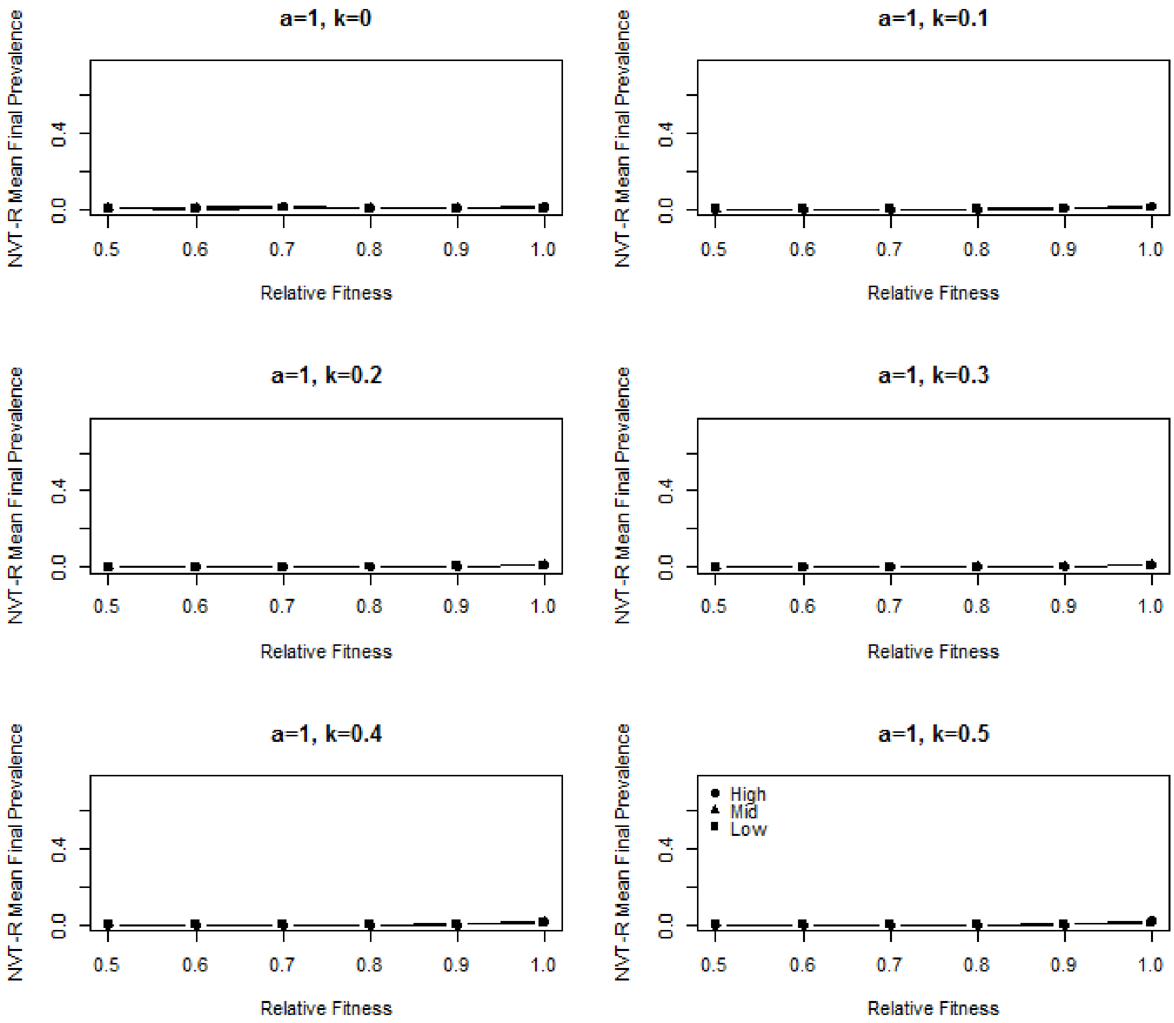

Patrick K. Mitchell, Marc Lipsitch, William P. Hanage. 2015 Carriage Burden, Multiple Colonization, and Antibiotic Pressure Promote Emergence of Resistant Vaccine Escape Pneumococci. *Phil. Trans. R. Soc. B.* **370** doi: 10.1098/rstb.2014.0342

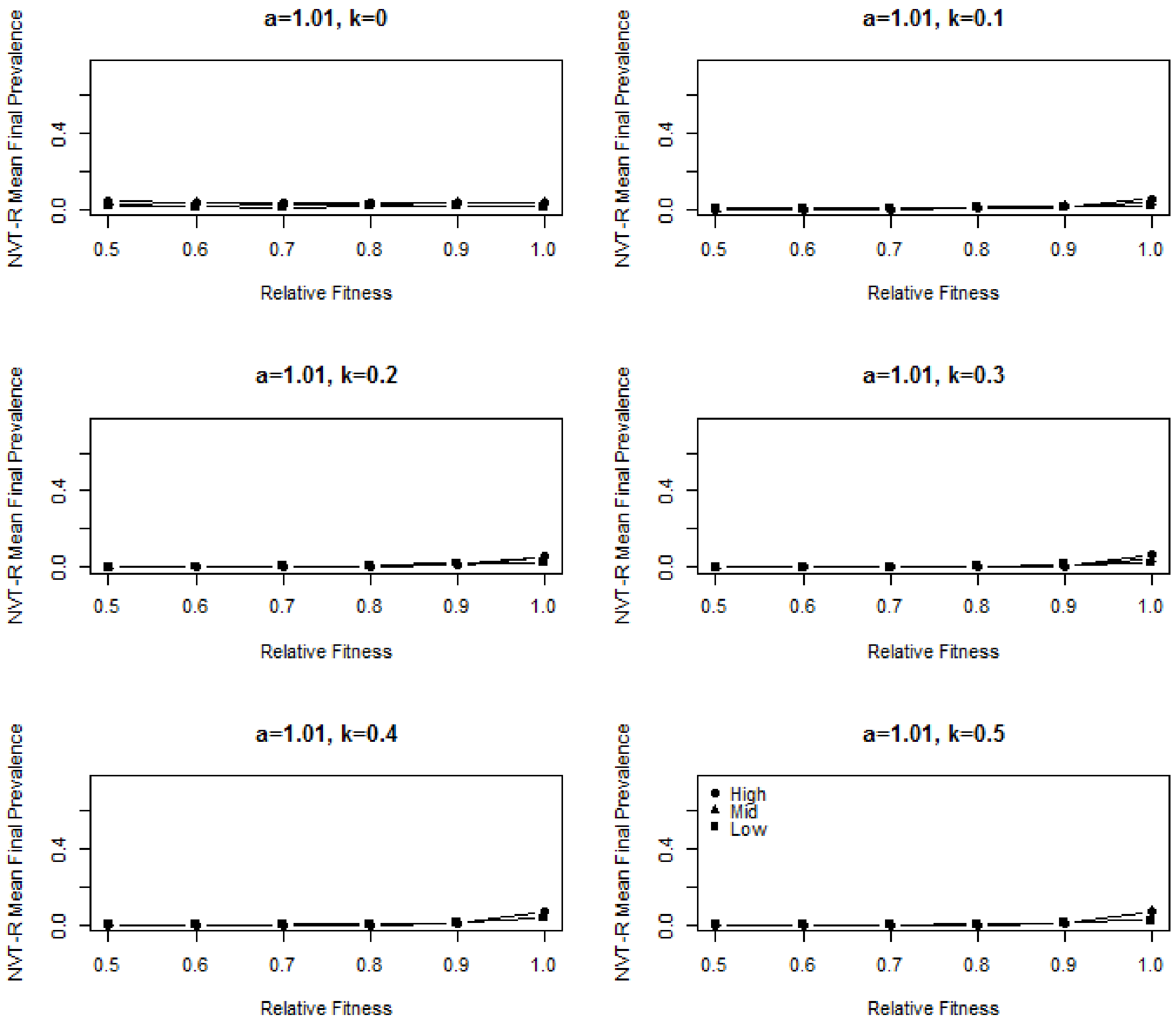

Patrick K. Mitchell, Marc Lipsitch, William P. Hanage. 2015 Carriage Burden, Multiple Colonization, and Antibiotic Pressure Promote Emergence of Resistant Vaccine Escape Pneumococci. *Phil. Trans. R. Soc. B.* **370** doi: 10.1098/rstb.2014.0342

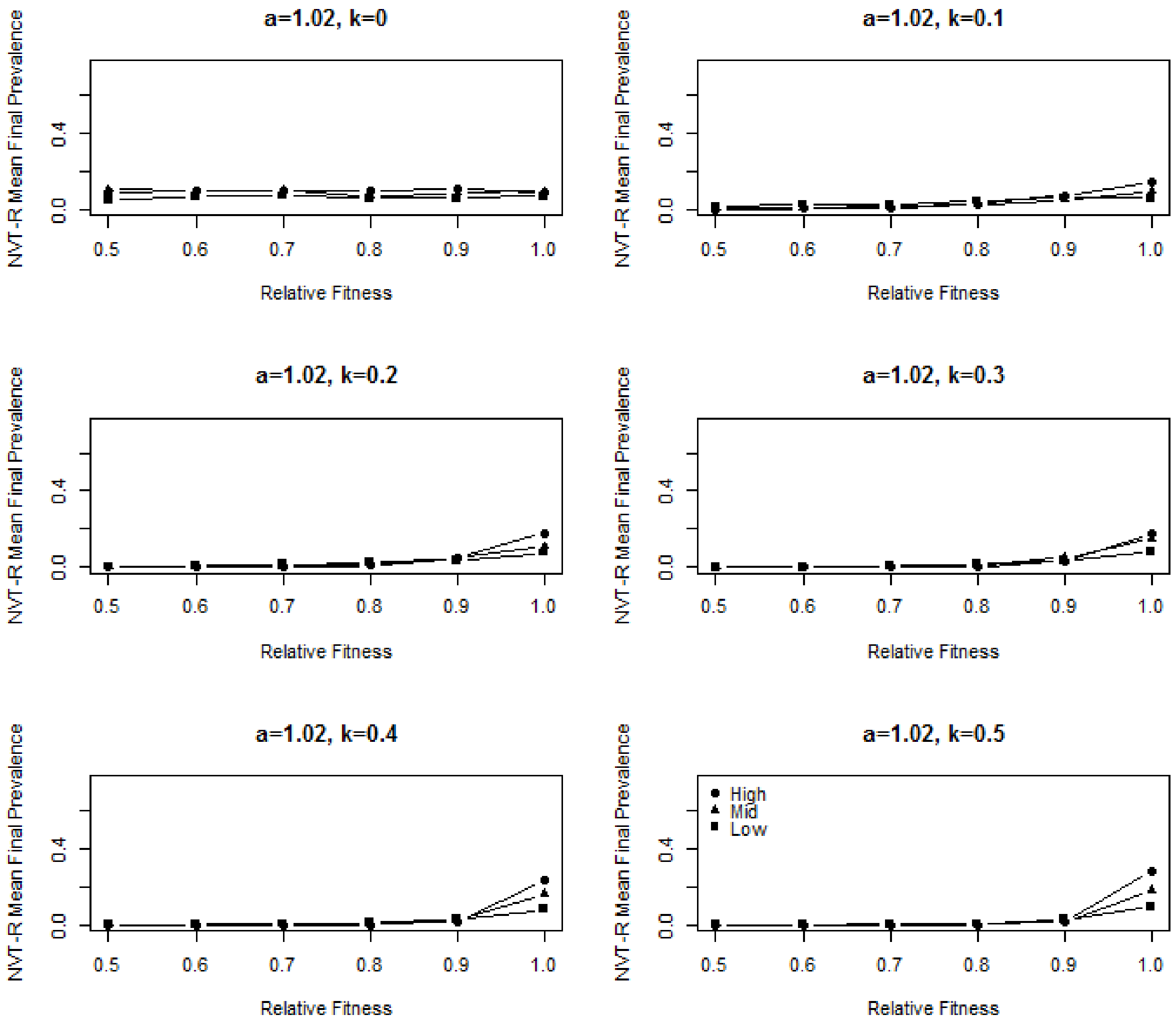

Patrick K. Mitchell, Marc Lipsitch, William P. Hanage. 2015 Carriage Burden, Multiple Colonization, and Antibiotic Pressure Promote Emergence of Resistant Vaccine Escape Pneumococci. *Phil. Trans. R. Soc. B.* **370** doi: 10.1098/rstb.2014.0342

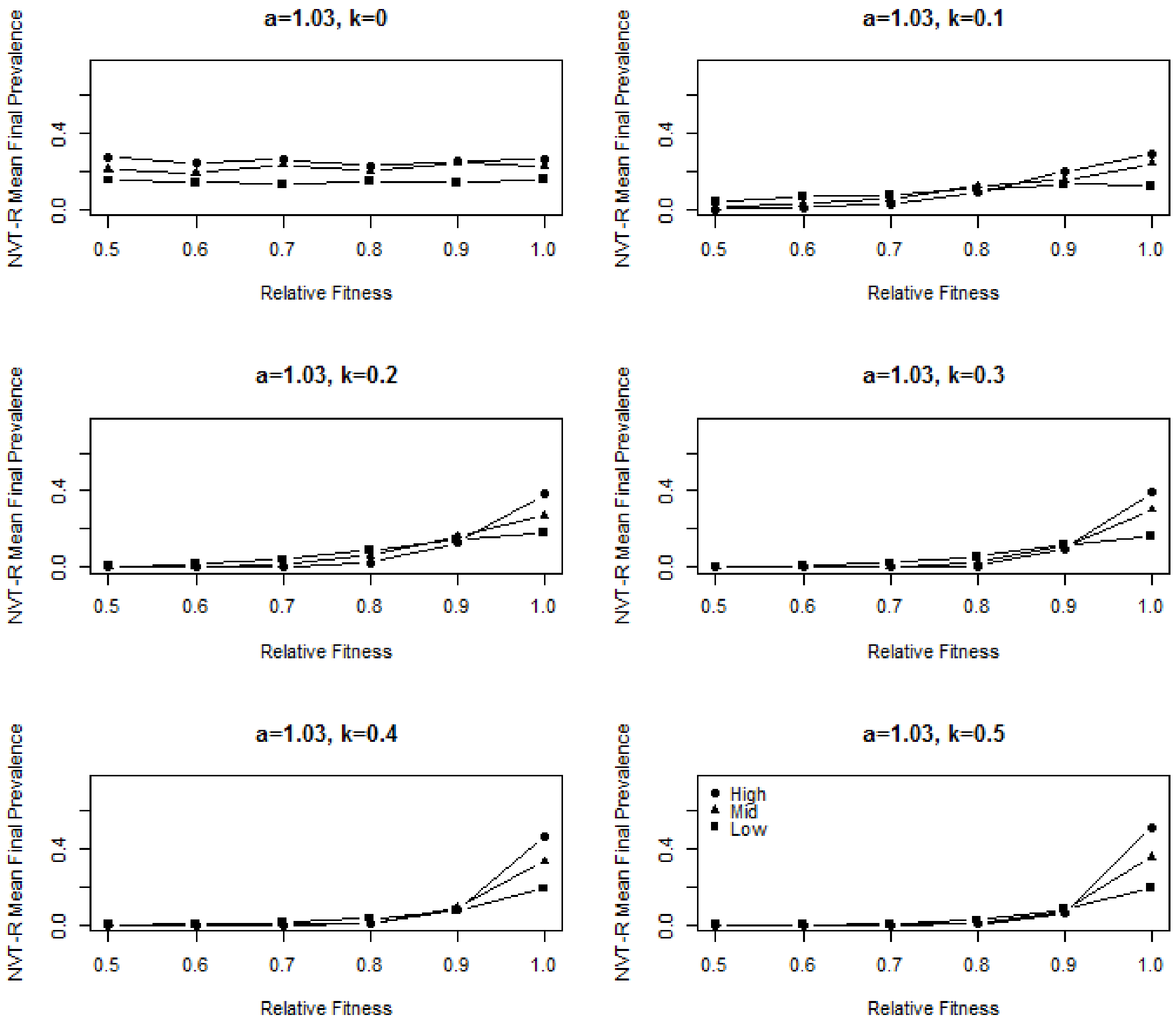

Patrick K. Mitchell, Marc Lipsitch, William P. Hanage. 2015 Carriage Burden, Multiple Colonization, and Antibiotic Pressure Promote Emergence of Resistant Vaccine Escape Pneumococci. *Phil. Trans. R. Soc. B.* **370** doi: 10.1098/rstb.2014.0342

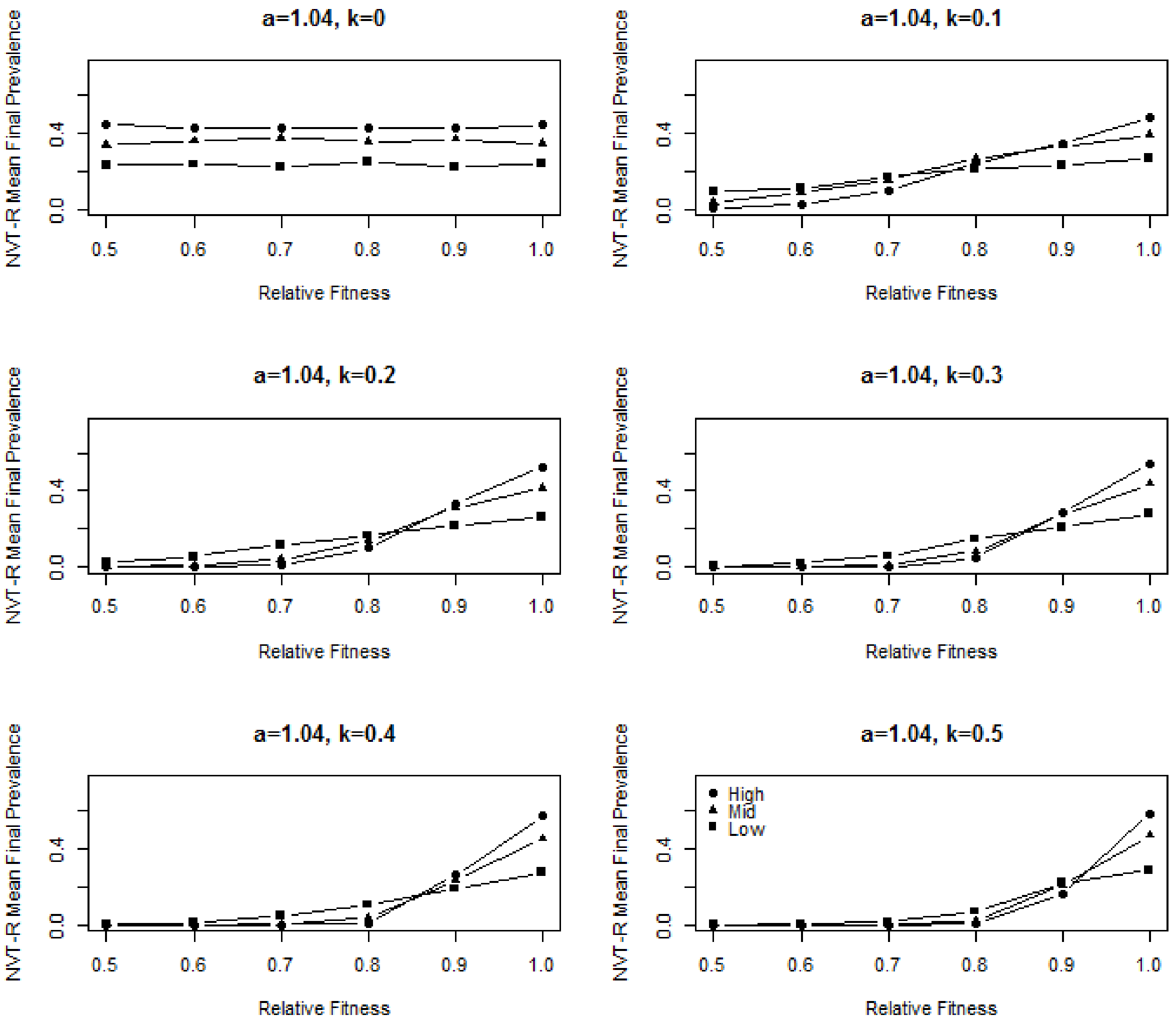

Patrick K. Mitchell, Marc Lipsitch, William P. Hanage. 2015 Carriage Burden, Multiple Colonization, and Antibiotic Pressure Promote Emergence of Resistant Vaccine Escape Pneumococci. *Phil. Trans. R. Soc. B.* **370** doi: 10.1098/rstb.2014.0342

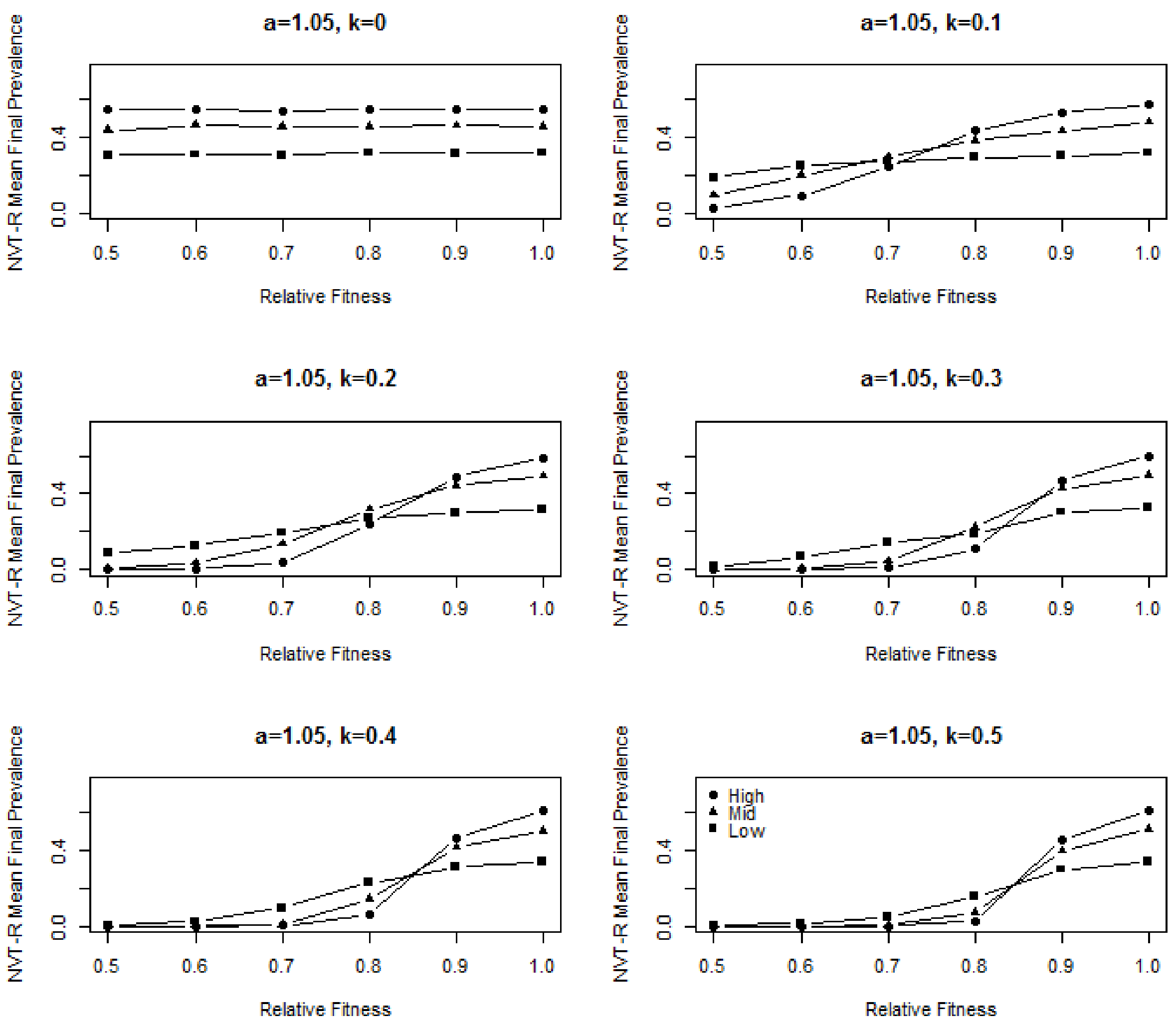

Figure S5: Mean final prevalence of NVT-R strain across values for relative fitness of resistant strains, with relative fitness defined as the ratio of transmission of a resistant strain versus a sensitive strain from a dual colonized host. Each panel contains the results for each of the three carriage burdens for a specific combination of antibiotic pressure (a) and dual carriage allowance (k).
